# Supplementary material for: Human resources challenges in the management of diabetes and hypertension in Mozambique
Source: PLoS One. 2024 Mar 29;19(3):e0297676. doi: 10.1371/journal.pone.0297676 (PMC10980202; doi:10.1371/journal.pone.0297676)
Supplement: Attachment — Submitted filename: Response to Reviewers.pdf [file pone.0297676.s001.pdf]

Response to the PLOS ONE Review Panel

Dear PLOS ONE Academic Editor,

This is to address the comments by reviewers to our manuscript "Human resources challenges in the management of diabetes and hypertension in Mozambique", submitted to your journal for publication.

We present a point-by-point response to the reviewers' comments below:

Reviewer Comments:

Reviewer #1

Overall

An interesting paper dealing with a relevant health issue. One may challenge the design of the study that incorporated components that were not purely qualitative. The theoretical framework was not clearly stated nor the data management. The results were not adequately presented.

Introduction

In the first paragraph, one might challenge that the references used are outdated. Could the authors use newer publications on the issues raised?

Answer: Thank you for raising this issue. The first paragraph of the introduction was revised to include up-to-date information.

Methods

Study design

One might challenge the place of document review and the observation that render the study not purely qualitative. I would suggest to use the information gathered from these two approaches only for the description of the study setting.

Answer: Thank you very much for this suggestion. Document review and observations were disregarded as data collection techniques and its content incorporated into study setting as suggested.

Theoretical framework

The methodological orientation was not clearly stated.

Answer: Thank you for highlighting this issue. The text was revised to specify the methodological approach used.

Study population

The justification of the sample size was not stated. Nor the number of the persons invited for the study. What was the response rate?

Answer: Thank you for this question. Lines 171 to 179 were re-wrote to clarify the rationale for the selected sample, number od persons invited and the response rate.

Data collection

The experience of the five researchers in qualitative research is not expressed.

The duration of the interview was not stated. What guided the authors to close the study? Data saturation?

Answer: Thank you! The above questions are now clarified and the revised text can be seen in lines 162 and 163, and from line 167 to 170 of the revised manuscript.

Data analysis

The role of the researchers in the data coding was not expressed. It is not clear how many persons did the data coding. Did the researchers participate in data coding? How did the authors acted to ensure the trustworthiness of their findings? Did they did member checking or peer review?

What kind of thematic analysis did the authors use?

Answer: The role od the researchers, coding details and type of thematic analysis used have been described in lines 214 and 225 to 228.

Results

Identification of the themes

It seems that the pre-identified themes are the same with that emerged from the interviews. Was there none deviation from what was described before the data collection?

Answer: There were no emerging themes during the coding process.

In the table 2, some themes had only one sub-themes, could the authors reconsider the grouping of the themes?

Answer: Thank you for your suggestion. This was partially accommodated since one of the themes was not easy to merge due to content and nature differences compared to the other within the same domain.

Presentation of themes

If one may encourage thick description, I would discourage to include in-text citations of references in this part.

Answer: Thank you very much for this suggestion. Since document review information was rearranged most of the in-text citations were removed as suggested.

References

For those in other languages than English, put their titles in English in square brackets.

Answer: Thank you very much for this suggestion. All other languages references were adequately translated and placed accordingly.

Reviewer #2

The manuscript is very well written with a clear abstract, introduction, methodology, results and discussion. The study objects have been answered diligently and conclusions match the results.

The grammar is excellent and easy to follow. Authors should correct one spelling error in line 440, replacing "by" to "buy.

Answer: Thank you very much for your encouraging comments and for highlighting the typo in line 440, which was corrected accordingly.
